# Evolutionary Features in the Structure and Function of Bacterial Toxins

**DOI:** 10.3390/toxins11010015

**Published:** 2019-01-03

**Authors:** Raj Kumar, Thomas M. Feltrup, Roshan V. Kukreja, Kruti B. Patel, Shuowei Cai, Bal Ram Singh

**Affiliations:** 1 Botulinum Research Center, Institute of Advanced Sciences, Dartmouth, MA 02747, USA; tfeltrup@gmail.com (T.M.F.); rv307@yahoo.com (R.V.K.); kruti.vasa@gmail.com (K.B.P.); 2 Department of Chemistry and Biochemistry, University of Massachusetts, Dartmouth, MA 02747, USA; swcai@aol.com

**Keywords:** botulinum toxin, evolution, snare proteins, ADP-ribosylation, bacterial toxin, anthrax toxin, cholera toxin, gluzincin clan

## Abstract

Toxins can function both as a harmful and therapeutic molecule, depending on their concentrations. The diversity in their function allows us to ask some very pertinent questions related to their origin and roles: (a) What makes them such effective molecules? (b) Are there evolutionary features encoded within the structures of the toxins for their function? (c) Is structural hierarchy in the toxins important for maintaining their structure and function? (d) Do protein dynamics play a role in the function of toxins? and (e) Do the evolutionary connections to these unique features and functions provide the fundamental points in driving evolution? In light of the growing evidence in structural biology, it would be appropriate to suggest that protein dynamics and flexibility play a much bigger role in the function of the toxin than the structure itself. Discovery of IDPs (intrinsically disorder proteins), multifunctionality, and the concept of native aggregation are shaking the paradigm of the requirement of a fixed three-dimensional structure for the protein’s function. Growing evidence supporting the above concepts allow us to redesign the structure-function aspects of the protein molecules. An evolutionary model is necessary and needs to be developed to study these important aspects. The criteria for a well-defined model would be: (a) diversity in structure and function, (b) unique functionality, and (c) must belong to a family to define the evolutionary relationships. All these characteristics are largely fulfilled by bacterial toxins. Bacterial toxins are diverse and widely distributed in all three forms of life (Bacteria, Archaea and Eukaryotes). Some of the unique characteristics include structural folding, sequence and functional combination of domains, targeting a cellular process to execute their function, and most importantly their flexibility and dynamics. In this work, we summarize certain unique aspects of bacterial toxins, including role of structure in defining toxin function, uniqueness in their enzymatic function, and interaction with their substrates and other proteins. Finally, we have discussed the evolutionary aspects of toxins in detail, which will help us rethink the current evolutionary theories. A careful study, and appropriate interpretations, will provide answers to several questions related to the structure-function relationship of proteins, in general. Additionally, this will also allow us to refine the current evolution theories.

## 1. Introduction

Bacterial toxigenesis is a major mechanism by which pathogenic bacteria produce diseases. They produce two kinds of toxins, lipopolysaccharides and protein toxins. Lipopolysaccharides are cell-associated toxins released after disruption of the cell (endotoxins), whereas protein toxins are synthesized inside the cells and then released to the target cells (exotoxins) [1]. 

Bacterial toxins, produced by bacteria primarily as virulence factors, are the most powerful poisons produced in the nature, and are known to retain a very high level of activity at high dilutions (highly potent molecule; [2]). These molecules are capable of performing some of the most remarkable tasks, such as formation of amazing nanomachines, specific targeting, learning and utilizing cellular processes, or modifications of cellular components. Additionally, the intoxication process itself involves very sophisticated steps. These unique qualities generate immense interest for biochemists, biotechnologist, protein engineers, pharma scientists, and medical professionals to utilize these molecules for better understanding of biochemical processes or to design future medical treatments. Despite years of investigation in this field, bacterial toxins still offer to raise several surprising questions and discoveries [3,4].

Bacteria are ubiquitous and utilize bacterial toxins as their weapon of choice in the arms-race. Bacterial toxin classification is primarily based on their structural and functional groups, as well as their mode of action. These molecules function in multiple ways, such as inhibition of protein synthesis (e.g., diphtheria toxin), destruction of cell membranes (e.g., *E. coli* hemolysin), activating secondary messenger (anthrax edema factor or cholera toxin), immune system activation (*S. aureus* super antigens), septic shock (bacterial endotoxins), or acting as an enzyme (tetanus or botulinum toxin) (Table 1). Based on the target of bacterial toxin action the following specific groups can be identified:

(a) Toxins targeting actin cytoskeleton components.
(b) Toxins targeting ubiquitin and ubiquitin-like signaling.
(c) Toxins targeting cell translational machinery.
(d) Toxins affecting secondary messengers and signaling components.
(e) Toxins disrupting the membrane integrity.
(f) Toxins with enzymatic activity.
(g) Toxins targeting DNA and inducing endoplasmic reticulum stress.

There is conclusive evidence available for the pathogenic role of various bacterial toxins in bacterial diseases. It is still a mystery, however, as to why certain bacteria produce such potent molecules while others do not. The production of toxin by a bacterium may not have anything to do with its survival, but instead they provide a particular niche in the adaptation to an uncongenial environment. Another related question would be why only a few strains are toxigenic while most others are not (even if they are phenotypically identical but unable to produce toxin).

Structurally, bacterial toxins seem to display some unconventional strategies. For example, toxins may have structurally and functionally identical domains but containing different binding domains (Diphtheria toxin and Pseudomonas ExoA), or could have similar binding domains but different catalytic domains (Clostridial toxins). These observations suggest a possible recombination in toxin evolution involving HGT (Horizontal Gene Transfer), and possibly insertion-deletion processes (gene-shuffling). These processes allow bacteria to find, or modify, their mechanisms to enter the target cells.

Existence of diversity in bacterial toxins poses a major challenge in defining the infection of host cells by the toxins. Other challenges include specific recognition of host cells, their toxin receptors, and determination of the mechanism of action. In this review, we are presenting a brief summary of some of the unique aspects of bacterial toxins covering structural, functional and evolutionary aspects.

## 2. Toxin Structures and Their Role in Defining Macromolecular Structure and Functions

### 2.1. Induced Folding of Bacterial Toxins

The previously held dogma of proteins dictated that a well-folded protein had a suitable binding site for its substrate and required no refolding. While this idea may work for many proteins, as researchers begin to observe more protein structures with spectroscopic techniques and X-ray crystallography, it is becoming apparent within the last decade that structural disorder in the proteome is far more common than previously believed [22,23,24,25]; it is estimated that approximately 1/3 of the human proteome, and a significant portion in other eukaryotes, is composed of fully, or partially, disordered proteins [22,26]. There are two broad classifications of the extent of structural disorder in the proteome—fully disordered proteins and proteins with disordered regions or domains (partially disorder proteins). Fully disordered proteins contain no secondary or tertiary structure and exhibit no equilibrium state [27,28,29]. To maintain this disordered state, the fully disordered proteins contain a high, typically negative, global charge along with a low mean hydrophobicity [24,29,30,31]. Together these features (global charge and hydrophobicity) introduce flexibility into the molecule. To adapt to changing cellular defenses, it makes sense that bacterial toxins would incorporate regions or domains of disorder within their structure in order to expand their longevity, substrate recognition speed (turn-over number or catalytic efficiency), and substrate binding affinity [32]. 

Disorder in a protein, whether global (unstructured, molten globule), such as an IDP (Intrinsically Disorder Protein), or local (e.g., loops or evolutionary selected regions), such as an IDR (Intrinsically Disorder Region), can play a very critical role in the protein function. This disorder can impact important protein characteristics such as stability within a cell and how the protein targets and binds its substrate [33]. The presence of disorder in a molecule introduces a very interesting concept of induced folding. When a flexible molecule interacts with its substrate or due to molecular crowding, the interactions introduce some structural changes in both, which is termed as induced folding. The induced folding allows proteins to overcome steric restrictions, have both high specificity and affinity towards its target, with a potentially large interaction surface, and yet maintain fast association/dissociation rates [34]. The increased structural plasticity of these proteins allows for a one-to-many style of interaction in which a single protein can interact with many targets [34,35,36]. This section will examine induced folding in proteins and the significant advantages it holds over conventional folding in proteins without IDPs. 

### 2.2. How Disorder Can Impact Substrate Binding

While some proteins maintain a disordered state throughout their functions, many undergo induced folding, marked by an unstructured-to-structured transition [34,37]. Induced folding is a key component of the function of a disordered protein or a protein segment in which the protein gains more typical secondary structure upon binding with a target [28,34]. This type of folding can augment the affinity of the protein to its target by providing precise control over the binding processes and protect certain regions of interaction to help modulate activity in response to different molecular targets [33,34]. As part of this mechanism, the unstructured protein or a protein segment in an unstructured conformation initially associates weakly with a target. During the initial association with the target, the appropriate conformation of the initially unstructured protein (or segment) is selected and its final structured conformation is obtained that more strongly binds to its target [38].

The common appearances of these IDPs and IDRs in cell signaling and regulatory pathways mark a clear favorability towards a one-to-many binding strategy within these pathways [27,33,37,39]. As mentioned previously, the ability of an IDP to bind to several different targets (one-to-many binding) decreases the energy demands on a cell in which it only needs to produce one protein to perform multiple functions instead of multiple proteins [40]. Since many cellular functions are tightly regulated and are both activated and deactivated quickly, a rapid turnover of proteins controlling these functions is required [33]. The IDPs offer a certain degree of instability in which they can be rapidly produced with low demands for folding, and likewise are rapidly degraded once the cellular response is no longer necessary [33].

One of the other benefits to a flexible structure is a larger capture radius of the IDP. This larger capture radius allows for the protein to find its target at a greater distance, such as a bacterial toxin searching for its target in an extra-cellular or intra-cellular matrix; however, this capture is with an initially weak interaction [38,41]. The ability to find its target in this manner is a significant advantage in cell signaling and regulatory pathways, despite forming only weak initial interactions [41]. This initial anchoring of the IDP to its target is subsequently followed by a disorder-to-order folding as the protein-target interaction proceeds [41].

An interesting adaptation to the proposed fly-casting mechanism by Shoemaker et al. [41], was previously described for botulinum neurotoxin [42]. In the case of the light chain of botulinum neurotoxin type A (LCA), the unbound LCA exists in a pre-imminent molten globule state characterized by a flexible global structure [43]. This flexibility and disorder allow for the increased capture radius as described by Shoemaker et al. [41]. However, the LCA does not follow an expected disorder-to-order transition upon binding to its substrate, SNAP-25. Instead, the LCA appears to become less structurally ordered upon binding to its target [42]. This unique case of induced refolding does not result in a more ordered structure and could be a key factor in the uncommon specificity, substrate length requirements, and longevity of botulinum neurotoxin [42]. A relatively more flexible protein would avoid/delay degradation inside the cells and increase its survival [44]. Proposed hypotheses for BoNT (Botulinum Neurotoxin) survival include Tyr-phosphorylation [45], increased resistance to ubiquitination [46,47], palmitoylation of cysteine residues [48], and S-nitrosylation [48]. One possible explanation would be that each of these pathways is affected by the varying degree of flexibility of the LCs and the longevity differences between serotypes may be due to the effect on these pathways in which the structural parameters play an important role [49].

Bacterial toxins have an ability to utilize disorder in their structures to undergo induced folding to operate. This flexibility may play a similar role in bacterial toxins as in other IDPs, allowing toxins to avoid cell regulation pathways and remain active in the cell for longer period of time, or exploit the plasticity of their structures to overcome intracellular hindrances [24,32]. In the next section, we present an overview of bacterial toxins which contain, and utilize, disorder in their folding to perform their functions.

### 2.3. Toxins Containing Disordered Regions in Their Native State

With many diseases being caused by disordered proteins in humans [26,35,50,51,52], it is not surprising that bacterial toxins have exploited structural disorder in their structure to enhance the ability to penetrate and damage healthy cells. Structural disorder within toxins has been well characterized and summarized previously [49], and shows toxins utilizing regions of disorder ranging from short stretches within domains (such as in colicin-E9 and calmodulin-sensitive adenylate cyclase), all the way up to larger stretches encompassing much of the entire structure (such as bifunctional hemolysin/adenylate cyclase). The role of this disorder can include targeting and binding with a substrate or receptor, membrane translocation, and/or exposure of the active site. Induced folding of intrinsically disordered regions, domains, or entire structures of bacterial toxins is not uncommon and typically follows a disordered-to-ordered transition upon binding [35,53]. This initial disorder and structural flexibility may be a key factor in allowing bacterial toxins to associate with their targets with high specificity by targeting longer substrate regions, maintaining their activity over extended time periods, and overcoming cellular defenses. Structural disorder and induced folding may also be a key component of the evolution of bacterial toxins to improve their effectiveness through molecular mimicry and adaptation [35,54]. In molecular mimicry, proteins (and toxins) would mimic the structure of a host protein to both enhance binding, and perhaps delay or avoid degradation by the host cell [35]. IDPs evolve rapidly through expanding repeat regions of disorder and can adapt and diversify their structure/function [55]. Flexible proteins and regions would provide a significant benefit to bacterial toxins with the ability to mimic host cell proteins with greater ease and rate of evolution. 

Virulence factors secreted by pathogenic bacteria are examples of IDPs and contain a repeat in toxin (RTX) motif, generally at the C-terminal end of the structure [56]. Many of these RTX motifs are involved in the host-pathogen interactions such as pore-forming, protease activity, lipase activity, and others [56,57,58]. Most of these pathogens with RTX motifs require calcium to perform their biological activity. In the absence of calcium, these proteins exist as IDPs, however, when bound to calcium, these proteins undergo a disorder-to-order transition with a notable decrease in net charge [56]. Motifs such as the RTX motif are examples of bacterial toxins overcoming host cell defenses. 

## 3. The Uniqueness of Enzymatic Function

The virulent factors of bacterial toxins can fall into many families. For example, ADP-ribosylating exotoxins have the virulent factors with ADP-ribosylating enzymatic activities whichinclude diphtheria toxin, cholera toxin, C2 and C3 toxin of *C. botulinum.* In this section, we are focusing on the enzymatic function, and more specifically on metalloproteases activity. 

*Bacillus anthracis* and *Bacteroides fragilis* are the bacteria that produce toxins belonging to metalloprotease family. *B. fragilis* toxin (BFT) is secreted by enterotoxigenic *B. fragilis*. It contains HEXXH, and is a Zn-dependent metalloprotease [59]. As many other metalloproteases, BFT is produced as a zymogen (proenzyme), requiring the removal of N-terminal pro-domain by an unidentified bacterial protease to generate a mature active toxin. It targets the extracellular portion of E-cadherin on intestinal epithelial cells, leading to a series of cellular signaling cascade, and resulting in production of proinflammatory cytokine, IL-8 [59]. BFT has structural homology to eukaryotic ADAM (A Disintegrin And Metalloprotease) metallopeptidases [60], and is the only established toxin to date for *B. fragilis*, suggesting that BFT is derived from a mammalian adamalysin/ADAM xenolog that was co-opted by *B. fragilis* through horizontal gene transfer from a eukaryotic cell to a bacterial cell [60,61].

Anthrax toxin is produced by *B. anthracis*. Anthrax toxin is an A-B type toxin, consisting of three parts: the receptor mediated binding domain, protective antigen (PA), and two enzymatic domains, lethal factor (LF) and edema factor (EF) [62,63]. The PA plays an important role in forming pores on endosomal membrane triggered by low pH through conformational changes. EF is a calcium and calmodulin-dependent adenylate cyclase, converting ATP (adenosine triphosphate) to cAMP (cyclic adenosine monophosphate), resulting in the elevation of intracellular cAMP level, and leading to edema. LF is a zinc dependent metalloprotease, targeting mitogen-activated protein kinase kinases (MAPKKs), and resulting in apoptosis [64]. The LF of anthrax toxin contains the signature HEXXH zinc binding motif and can be grouped into the same gluzincin clan as clostridial neurotoxins. Sequence and structural analysis revealed that Mlc (makes large colony) titration factor A (MtfA) of *E. coli* is also a prototypical zinc metallopeptidase (gluzincin clan) and is related to LF of anthrax toxin and clostridial neurotoxins [65]. While there is a low sequence homology between MtfA and LF overall, the zinc biding motif is highly conserved. Further structural alignment of MtfA and LF revealed that the catalytic residues (HEXXH + E + Y) are all harbored in a highly conserved core corresponding to a helix containing the HEXXH motif and followed by a 3_10_-helix-turn-helix containing a YX6E motif in their sequence. Another related toxin protein with highly conserved zinc binding region is Mop (modulation of pathogenesis) of *Vibrio cholera*. Interestingly, the tyrosine which is essential for catalysis (Y375) in botulinum neurotoxin type C1 is also placed at a similar location as in MtfA LF and Mop as observed from their respective crystal structures [65]. The similarity of catalytic residues’ arrangement between MtfA-like domain and botulinum neurotoxin is intriguing despite their different evolutionary origins (see below). 

**Figure 1:**
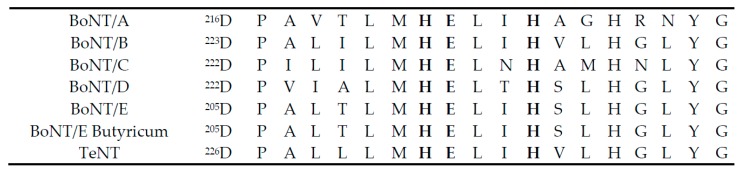
The HEXXH Zn^2+^ binding motif in the conserved hydrophobic region of clostridial neurotoxins [73].

**Figure 2:**
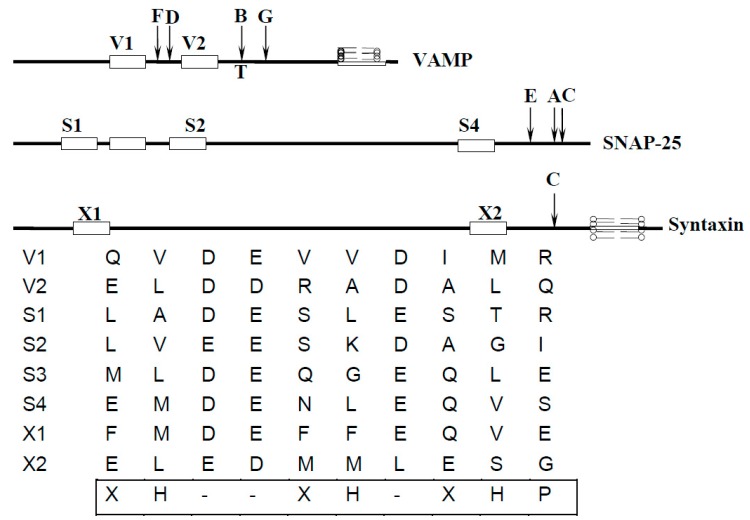
Positions of SNARE motif in three substrates responsible for the target specificity of clostridial neurotoxins [69]. The motif consists of nine residues which is common to all three substrates: hydrophobic residue (H), Asp or Glu residue (-), polar residue (P), and any residue (X).

The most important and versatile zinc-metalloproteases are BoNTs and TeNT (Tetanus Neurotoxin) with a short zinc binding motif HEXXH (Figure 1). They belong to the gluzincin clan [66]. However, unlike all other metalloproteases (including gluzincin clan), BoNTs and TeNT have a high selectivity for their substrates. In contrast to other metalloproteases, clostridial neurotoxins specifically target only SNARE proteins, while the substrate selectivity for most other metalloproteases are not that specific. It has been hypothesized that there are unique enzyme-substrate interactions between toxins and their substrates, and there is an exosite-dependent endopeptidase activity of the catalytic domain of the toxins [67]. All clostridial toxins cleave their targets at different cleavage sites, with the exception of BoNT/B and TeNT, which cleave the same site in VAMP. The highly selective cleavage sites of clostridial toxins are unusually unique among the endopeptidases as they target: 1) a specific peptide bond in their substrate; and 2) only three SNARE proteins (VAMP, SNAP-25, and syntaxin) involving exocytosis. It is believed that this selectivity is due to the presence of a highly conserved SNARE motif [68] in their targets [69]. As shown in Figure 2, the SNARE motifs include V1 and V2 in VAMP, S1 through S4 in SNAP-25, and X1 and X2 in syntaxin. It is predicted that these motifs are adopted as a helical conformation with three negative charges contiguous to a face formed by hydrophobic residues [69]. Short peptides corresponding to the SNARE motifs showed inhibition activity against different BoNT serotypes; antibodies against those SNARE motif peptides showed cross-reactivity among the three substrates, and the inhibition of each other’s proteolytic activity [69,70]. While BoNT/B and TeNT cleave the same peptide bond (Gln-Phe), they show different affinity to V1 and V2: TeNT, andBoNT/D, need the V1 motif, whereas BoNT/B (like BoNT/G) has a high affinity to V2. BoNT/F, on the other hand, recognizes the V1 and/or V2 motifs [69,70,71]. VAMP cleaved by TeNT is accelerated by nerve stimulation. However, BoNT/B, despite cleaving the same site as TeNT, does not require any stimulation [72]. This suggests that V1 and V2 domains may not be exposed under the same physiological conditions; before nerve activity, V1 is protected in protein complex, while V2 is exposed [72]. For the effective cleavage of SNAP-25, the C-terminus of SNAP-25 (containing S4 motif) is required for both BoNT/A and /E. The three N-terminal motifs (S1, S2, and S3), however, can partially be substituted for a modified S4 motif [68]. Notably, BoNT/C is the only toxin which cleaves two substrates: SNAP-25 and syntaxin.

The required SNARE motif(s) within their respective substrates for cleavage suggests that the hydrolysis of the peptide bond by clostridial toxins may be mediated by binding of those required SNARE motifs with the complementary sites presented on toxins. This exosite-controlled hydrolysis was hypothesized in late 1990s based on the biophysical studies on the binding between substrate and toxin [67,74]. The interactions of BoNT/A, /E, and /C with SNAP-25 have been thoroughly examined in order to understand the catalytic mechanism. Through co-crystallization studies of BoNT/A-SNAP-25 complex, two exosites have been identified in BoNT/A [75]. Structural and sequence analysis also helped to propose the potential exosite(s) on the light chain of botulinum neurotoxin type B, D, F, G, and tetanus neurotoxin that recognize(s) SNARE recognition motifs of VAMP [76,77,78,79]. Identification of exosites on clostridial neurotoxins not only helps elucidate the specificity of these toxins and the mechanism of catalysis, but also provides additional target sites for designing novel inhibitors against these deadly toxins. In addition, it provides the fundamental information on enzyme substrate recognition. 

## 4. Symphony of Enzyme-Substrate Interactions for Harmonious Function

As mentioned previously, bacterial toxins are potent virulence factors that disrupt the cellular functions of the host by altering the activity of intracellular proteins through several unique strategies. In doing so, changes not only occur in the substrate, it influences the enzyme conformation as well., andresults in a very concerted effort to execute a precise function. Bacterial ADP-ribosylating toxins play diverse roles in pathogenesis by employing a strategy involving ADP-ribosylation of unique eukaryotic protein targets to disrupt important physiological processes, such as protein synthesis, signal transduction, etc. in the infected host cells. Examples of this strategy include diphtheria toxin (DT), pertussis toxin (PT), cholera toxin (CT), *E. coli* heat labile toxin (LT), pseudomonas aeruginosa toxin (ET) and botulinum C3 toxin [80]. 

DT and ET through the ADP-ribosylation of diphthamide in elongation factor 2 cause inhibition of protein synthesis. Through ADP-ribosylation of regulatory G proteins at Arg or Cys residues, CT, PT and LT interfere with signal transduction in human host cells thereby inhibiting actin polymerization. The family of C3-like ADP-ribosyl transferases are known to ADP-ribosylate a number of low molecular mass GTP binding proteins, interfering with small Rho GTPase-mediated signal transduction [81]. Although these toxins modify various target proteins and have low amino acid homology, they share similar overall three-dimensional structures and a NAD-binding pocket. A glutamate residue essential for catalytic activity is also conserved in all ADP-ribosylating enzymes, suggesting a common catalytic mechanism amongst these toxins [82].

The first toxin discovered to act via ADP-ribosylation is DT and it has been extensively characterized since then. DT is a 58 kDa, 535 residue protein secreted by *C. diphtheriae*. In humans this pathogen causes the disease diphtheria by damaging host cells. The structures of monomeric and dimeric forms of DT, its isolated catalytic domain and of dimeric DT bound to NAD, have been determined by X-ray crystallography [83].

Kinetic and binding studies have revealed that the transfer of ADP-ribose group to EF-2 from NAD is catalyzed by the DT. This transfer proceeds through an ordered sequence wherein the binding of C-domain to NAD forms a binary complex which results in its binding to EF-2. Binding of NAD has been shown to be an essential step in the catalysis, indicating that the binding of NAD induces significant structural changes in the functionally important C-domain for subsequent binding of the acceptor substrate [84] and was reflected in the crystal structure of diphtheria toxins bound to NAD [85]. The catalytic domain of DT consists of several hinge loops that endow it with the potential for flexibility. Residues 34-52 form a well-ordered loop which extends over the active site. Upon binding to NAD, this loop becomes disordered through subtle interactions between NAD with certain residues in the active site cleft allowing it to release from its ordered position over the active site. The NAD binding-induced structural changes, which subsequently help in the releasing of the active site loop, allowing subsequent stronger binding of ADP ribose acceptor substrate EF-2 to NAD [85]. In a similar fashion, ET also catalyzes the ADP ribosylation of EF2. Crystal structures of ET and its catalytic domain complexed with NAD have shown that the active-site loop of ET (similar to DT) exhibits similar conformational flexibility in the presence of NAD which plays an important role in its binding to the substrate [83].

Cholera toxin (CT), a major virulence factor produced by the *V. cholerae* bacterium, causes cholera, a serious diarrheal disease, by infecting the human intestine. The catalytic moiety A1 is an ADP-ribosyl transferase that catalyzes the covalent transfer of an ADP-ribose moiety from NAD to Arg201 of the signaling protein Gsα. This reaction, which is allosterically activated involving human ADP-ribosylation factors (ARF), resulting in a series of events that culminate in a dramatic efflux of ions and water from the epithelial cells into the intestinal lumen, generating the watery diarrhea characteristic of cholera [86].

Crystal structures of CT and CTA1:ARF6-GTP complex suggests that upon binding to ARF6-GTP, the active site loop in the catalytic domain which occludes that active site swings out or is released from the active site through subtle structural shifts in its vicinity. The conformational changes in the active site loop expose the active site residues implicated in the substrate binding and catalysis, allowing NAD to bind to the active site. Thus, it was observed that the binding of ARFs alters the catalytic domain of CT such that the toxin adopts a structurally different conformation to recognize the target G-protein substrate Gsα [87], and catalyzes its ribosylation.

The catalytic subunit (S1) of PT, produced by *B. pertussis*, demonstrates similar structural organization to the catalytic domains of other ADP-ribosylating toxins, such as the cholera toxin family. Pertussis toxin plays a central role in the etiology of whooping cough and evoking a protective immune response against it. The crystal structure of PT reveals that while the N-terminal domain, which is homologous to the other ADP-ribosylating toxins, contains a similar NAD binding site and the residues involved in catalysis, the differences in the C-terminal domain of S1 (involved in binding of G protein) explains its unique activation mechanism. PT catalyzes the ADP-ribosylation of Cys-352 of Gα_i_. The Cys residue at this position is absent in other Gα proteins. Analogous to the mechanism of ADP-ribosylating enzymes aforementioned, the crystal structure of PT bound to NAD has implied conformational changes in the active site loop upon binding to NAD, which aids in subsequent binding of the toxin to the heterotrimeric G proteins that function as the ADP ribose acceptor substrates for PT [80]. ADP-ribosylation of Gα_i_ proteins by PT resulting in the uncoupling of interaction and activation of Gα_i_ by the receptor, which, in turn blocks the GDP/GTP exchange effectively intertwine the Gα subunit in its GDP-bound heterotrimeric form [88]. Thus, it is observed that the active site loop which is positioned near the NAD binding site, and which is displaced upon binding of NAD, is conserved among several ADP-ribosylating toxins and plays a crucial role in substrate binding and catalysis. 

C3 exoenzymes, an ADP-ribosyl transferase protein, have a single domain organization comprising solely of a domain responsible for catalyzing the ADP-ribosyl transfer reaction. C3-like toxins modify Rho GTPases essentially by inactivating them which results in subsequent inhibition of the targeted signal transduction pathways. C3 bot, produced by a *C. botulinum strain*, ribosylates Rho A, RhoB, and RhoC by transferring the ADP-ribose moiety of NAD on to the amino acid Asn41. The crystal structure of C3 in complex with Rho demonstrates how the binding of NAD and Rho triggers an independent conformational change in the phosphate nicotinamide (PN) loop and the ADP-ribosylating turn-turn (ARTT) loops, respectively, and the critical role of the conformational flexibility of the ARTT loop in Rho binding. In addition, large conformational changes in RhoA upon binding to C3 were also observed. Thus, it was concluded that plasticity of both C3 and Rho is crucial for their interaction and efficient catalysis [89].

The most dramatic changes introduced in the structure of an enzyme and its substrate is observed in botulinum toxin. The catalytic domain of BoNT/A is a α/β protein with a zinc metalloprotease active site, in which zinc is bound deep inside a large cavity. Superimposition of the X-ray crystal structures of BoNT/A, B and E light chains (LCs) reveals virtually identical structures of their active sites [75,90,91]. However, unique substrate selectivity and cleavage site specificity demonstrated by the serotypes of BoNTs suggest that the enzyme’s active site cleft does not solely dictate the enzymatic action. The crystal structure of the catalytic domain of BoNT/A reveals the presence of four flexible loops that form the rim of the active site cleft and possibly participate in substrate binding [75,92,93]. Structural elucidation of the catalytic domain of BoNT/A in the presence of substrate SNAP-25 has provided an evidence for the presence of systematic step-wise substrate recognition and binding sites known as exosites (α− and β-exosites) that are remote from the active site [75]. Based on these observations, a model for substrate recognition has been proposed wherein the interaction of the amino-terminal helical region of SNAP-25 with the enzyme initiate substrate binding along a hydrophobic patch formed at the interface of four α-helices of the LC referred to as α-exosite. This is followed by binding of the C-terminus of at the β-exosite of BoNT/A LC. This interaction induces conformational changes in the active site pocket of the enzyme, facilitating the protease become competent for catalysis [75]. This multi-site binding and recognition strategy used by BoNT/A accounts for the extreme selectivity of this enzyme [94].

The catalytic domain of BoNT/A and the entire holotoxin have been shown to exist in a dynamically flexible and enzymatically active PRIME and molten globule states, respectively, at physiological temperature [43,95]. The higher intra-molecular mobility of PRIME state relative to its native state can be attributed to increased dynamics and provide necessary expansion of the protein core, thereby facilitating a very specific interactions with its substrate, SNAP-25, which is required for its optimum and selective enzymatic activity. This feature is assumed to play a vital role in the biological function of BoNT/A, especially in its intracellular toxic action. The dynamic role of such structure is not only significant to define its extreme specificity of the endopeptidase activity towards its substrate, it is also of tremendous utility in designing specific antidotes against botulism threats. An understanding of BoNT structure in physiological conditions is crucial to provide detailed mechanism involved in its action.

The mechanisms through which enzymes attain extraordinary reaction rates and specificity have long been of interest in biochemistry. Understanding the function of an enzyme on an atomic level has been revolutionized by high-resolution X-ray crystallography and to some extent by NMR (Nuclear Magnet Resonance) spectroscopy, resulting in generation of several data and related studies of structure–function relationships. The synergy between structure and dynamics is one of the important factors to the functioning of biological macromolecules. For optimum activity, stability of enzymes should be enough to retain their native three-dimensional structures, but with enough flexibility which will allow sufficient substrate binding, chemical reaction and product release. Conformational changes in the structure of these toxins upon substrate binding impart plasticity in the enzymes for optimal enzymatic activity. Bacterial toxins being the potent virulence factors form essential pharmacological tools to study the physiological functions of their eukaryotic targets. Advancements in understanding the structures of bacterial toxins have been valuable in deciphering their mechanisms of action, thereby providing us with molecular clues for further discoveries allowing the use of these toxins in cell biology and neurobiology. 

## 5. Other Protein—Protein Interaction of Bacterial Toxins

Part of the uniqueness of bacterial toxins is their stability inside the target cell (in general; Table 2). During its life inside the cells, these toxins not only interact with their substrate, but also interact with other proteins and affect the other cellular functions.

Ratts et al. [72] recognized a functional role for human heat shock protein 90 (Hsp 90) to be fundamentally critical in mediating refolding of the diphtheria toxin catalytic subunit [97]. Haug et al. [73] observed the involvement of Hsp90 in the translocation of enzymatic domain C2I from *C. botulinum* C2 toxin [98], an ADP-ribosylating G-actin protein. Hsp90 is involved with other bacterial toxins facilitating their entry from the lumen of endosomes, such as iota toxin [99] and anthrax toxin [100]. Most studies demonstrated an inhibitory effect of geldanamycin or radicicol alone, or together, act on Hsp90. A co-chaperone of HSP 90 is cyclophilin (CypA), which binds to the immune-suppressor protein cyclosporine A (CsA) which facilitates the cytosolic entry of the enzymatic domain of *C. botulinum* C2 toxin, *C. perfringens* toxin, and the *C. difficile* an actin-ADP ribosylating clostridium toxins [101,102]. The translocation of the fusion toxin LF (lethal factor) of anthrax and A domain of DT (LF_N_DTA) require Hsp 90 and CypA [100]. 

Along with HSP proteins, thioredoxin (Trx) plays a key role in the folding of proteins. The S-S bond is reduced by the thioredoxin reductase (TrxR)—thioredoxin (Trx) system leading to the detachment of the A domain (of A-B toxins) from the membrane during their translocation into the cell. This process releases the catalytic domain of A-B toxins, such as diphtheria, ricin, botulinum, and tetanus toxins into cytosol [97,103,104].

Many different bacterial toxins target Rho GTPases and ADP ribosylating factors which are responsible to control actin-based cytoskeleton. The Rho proteins (Rho, Rac, Cdc42) comprise of Ras super family of small GTPases which act as molecular switches in various signaling pathways and plays an important role in regulation of the actin cytoskeleton [105]. Glycosylating toxins inactivate the small GTPases (Rho and/or Ras) by the addition of glucose on a conserved Thr residue [106]. These glycosylating clostridial toxins include *C. novyi* (α-toxin), *C. sordellii* (lethal toxin), and *C. difficile* (toxin A and toxin B)**, and. Toxin B catalyzes glycosylation of Rho protein (Rho, Rac, cdc42), whereas lethal toxin modifies Rac, cdc42 and various Ras proteins such as Ras, Rap, Ral, which are involved in the regulation of the actin cytoskeleton. Furthermore, BoNT/A has been reported to target RhoB to promote accelerated RhoB degradation by proteasomes, causing blockage of actin cytoskeleton dynamics and obstructing release of acetylcholine [107]. One of the essential post-translational modifications inside the cells is the endogenous ubiquitination system. It is a reversible process playing crucial roles in protein degradation, host defense, signal transduction, and vesicular trafficking [108]. Botulinum toxins have been reported to be interacting with the cellular ubiquitination system. TRAF2 (TNF receptor associated factor 2), a crucial protein responsible for the ubiquitin process, has been shown to interact with BoNT/E and promotes its degradation [46]. However, BoNT/A avoids this pathway [109]. The presence of dileucine motif containing (EFYKLL) located near the carboxyl end (amino acid 427/428) of the endopeptidase domain (present in LCA, but not in LCE) involved in the plasma membrane sequestration, in which helps LCA to avoid ubiquitin-protease degradation pathway [110,111,112]. The differences in the interaction of ubiquitin protease system with BoNT explains the differences in longevity of BoNT/A and BoNT/E (to some extent). The wtLCA co-localizes with septin-2 and septin-7 of plasma membrane-associated septin clusters, interestingly through the di-leucine motif. Thus, interaction of LCA with septin–2 and septin-7 is very crucial for the long-lasting paralytic effect [112].

## 6. Evolution of Bacterial Toxins

Pathogenic bacteria have developed an array of sophisticated virulence factors in the process of evolution for survival. These factors allow them to invade, replicate, and colonize within an immune competent host. Most of the bacterial virulence factors generally target or evade the cellular processes to invade their host [113]. The level of complexity present in some toxins allows them to exert multiple functions, e.g., pore forming toxins and regulators of intracellular processes [114,115,116]. 

The specificity and uniqueness of bacterial pathogenesis generate several intriguing scientific questions: (a) origin of the protein toxin? (b) purpose of producing these toxins? (c) the rationale behind the diversity of these toxins? (d) is there any selective advantage to the bacteria? and (d) how are they able to target humans? Kumar and Singh [117] discussed some of these issues in detail. One thing is very clear that the conservation of basic intoxication processes (for example, A-B toxin) suggests the evolutionary learning processes which may be important for their survival and proliferation in natural environment. These learning processes are highly successful. During evolution, they learn some unique traits such as morphological adaptations (e.g., elongation, spore, biofilm or filament formation), growth characteristics, motility, toxicity and ability to replicate, and avoiding degradation in eukaryotic cells.

Bacterial exotoxins are carried by gene-encoding or phage-encoding, which converts them from avirulent strain to a pathogen [118]. Although they do target humans and mammals, these phages exist in high frequencies as free phages and in lysogenic bacteria. Additionally, their targets are evolutionary conserved pathways. These observations suggest that mammals are not their primary targets. In addition, HGT between eukaryotes and bacteria is rare. So, a question arises that if mammals are not their true targets then how do they invade humans? One explanation comes from the co-existence of bacteria and protozoa. This co-existence led to the evolution of antipredator strategies in protozoa or both. This leads to a widely accepted hypothesis that many pathogenic traits in bacteria were shaped outside the human-pathogen interactions. Humans may be innocent bystanders between the arm-race between protozoa and bacteria and virulence factors were developed inside the bacteria as an accidental virulence [119,120]. 

The prevailing paradigm of bacterial evolution is periodic modifications triggered by horizontal gene transfer. Additionally, all genes in the genome of bacterium will show a close relationship with their nearest ancestor. This paradigm holds true for most bacteria, but not for all. Furthermore, in addition to these major changes a bacterium is subject to spontaneous point mutations that can modify any gene in the genome. Later, because of the accumulation of these changes, bacteria can either retain a function or diversify itself to promote immune evasion. Similar to evolution of all forms, bacterial toxin phylogenetic roadmap comprises of the evolution of functional progenitor molecule, followed by adaptive processes. The adaptive processes are the evolutionary fine tuning which modify the catalytic efficiency, substrate specificity, altered interaction with target cells, and the host response. Commonly, blocks of genes move together. However, there are several insertion and exchange of genes between different phylogeny for different parts of genes, referred to as “mosaicism” or HGT. 

HGT can occur within a species, for example, hemolysin encoding lktA gene of *M. haemolytica* and for pathogenic *E. coli*. It may also occur between closely related species, for example, movement of Shiga toxin genes between *E. coli* and *S. dysenteriae*. Introduction of a new gene to other organism involves either complete or partial replacement of the incumbent gene. Using HGT *C. difficile* and *C. perfringens* are capable of converting a non-toxigenic strain to a toxin producer [121,122]. However, in most cases, this process involves transfer of totally new set of genes to the host genome. Additionally, it is possible that a new gene may be acquired by the organism which is already possessing a similar allele and both copies can be retained (paralogs or orthologs). Interestingly, bacteria are able to shuffle their virulence factors. They achieve this by localizing their multiple toxin-encoding genes on plasmids together with mobilizable part of the plasmid [123] Recombination events and sequence diversity helps them to develop a better armory. 

The diversity of domain, folding, specificity, and sequence divergence are important characteristics of bacterial toxins. One of their features is the existence of functionally complementary domains (target recognition, internalization and translocation, and intracellular biochemical effector), all with independent functions packed into a folding nanomachine. Therefore, there must be a role of structural elements in the evolution of bacterial toxins. Since proteins are not static molecules, there must be a subtle interplay between rigidity and flexibility of different domains, within the domains, and within various structural elements, which ultimately drives the evolutionary processes perhaps at a more fundamental level. Kumar and Singh [117] explained the importance of these elements in evolutionary processes.

*Clostridium botulinum* and *C. tetani* are ancient bacteria. Analyzing their sequences and study of their function suggest that the ancestor of neurotoxins may not be in *Clostridium* genus, but rather may have been from viral polyproteins [124]. Alternatively, as highlighted by Kumar et al. [125] that although there is very little similarity in the sequence outside the Clostridial Neurotoxin (CNT) family, it is possible to find an evolutionary correlation by establishing the relationship with similar structural and functional domains. Thus, two possibilities of BoNTs evolution are: BoNTs either evolved from a family of BoNT-like toxins, or BoNT genes may have spread to other organisms through HGT. Based on the observation of Cordes and Binford [126], it has been hypothesized the possibility of transfer of genes from eukaryotes to prokaryotes, and then integration into bacterial proteins through HGT [117]. 

The light chain of clostridial neurotoxins are metalloproteases with HEXXH motif in their active site, and more specifically, are classified into the M27 family under MA clan [127]. Would this come from the common ancient ancestors during the evolution? It is reasonable to hypothesize that the light chain of BoNT is derived from some other family of MA (Metalloprotease super family A) proteases. DasGupta hypothesized that the ancestors of those neurotoxins were not originally within the genus of Clostridium, rather derived from viral polyproteins [124]. This hypothesis has been supported by various new findings of BoNT homologs, such as Weiseella toxins in *Weissella oryzae* SG25 [128], BoNT/X in *C. botulinum str*. 111 [129]; BoNT/En in *Enterococcus faecium* str. DIV0629 [130]. Mansfield and Doxey [131] identified homologs of BoNTs in *Weissella, Entercoccus, and Chryseobacterium*. Even though these homologs are outside the Clostridial family, they establish the ecological role of Clostridium family and BoNT. However, the repertoire of BoNT-like genus outside *Clostridium* is quite narrow, and it is difficult to suggest a lineage that BoNT-like toxins first evolved from, as well as the direction of the relationship between M27 and other MA protease family [132]. The translocation domain of clostridial neurotoxins, on the other hand, shares some similar mechanisms as other AB type toxins. Through sequence alignment analysis, the sequence similarity and common motifs are identified between diphtheria T domain and an N-terminal portion of the BoNT translocation domain, suggesting that there is a distant relationship to the translocation domain between clostridial neurotoxins and the diphtheria toxin T domain [131]. Therefore, the translocation domain may be evolved through other pre-existing toxins with functionally similar protein domains. The heavy chain binding domains (both N-terminal and C-terminal) are present in all clostridial neurotoxins, and the BoNT-like toxin in *Weissella*. This suggested that the binding domains may exist in the early BoNT ancestor. 

**Figure 3:**
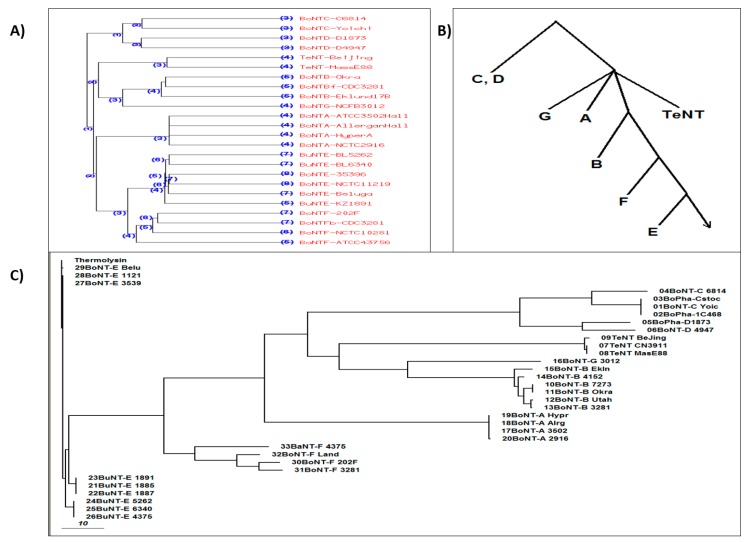
(**A**) The dendrogram of the molecular evolution tree, and (**B**) their rooted phylogenetic tree (bottom) for all 7 serotypes of botulinum and tetanus neurotoxins. (**C**) The predicted evolution tree. Results are based on the sequence alignment of full-length proteins [100].

The HEXXH motif is conserved in most monometallic metalloproteases, including clostridial neurotoxins. This suggests that the substrates are not necessarily limited to SNARE proteins from vertebrate for clostridial neurotoxins. The co-evolution of substrate and toxins could play a role in the unique specificity of clostridial neurotoxins [125,133]. Chang and Singh [133] established the phenogram of different serotypes and their subtypes of clostridial neurotoxins, suggesting that BoNT/E is farthest (the least sequence convergence) from BoNT/C and /D (the most sequence convergence), while BoNT/A and TeNT is in between (Figure 3; [133] Further analysis of the substrates of BoNT/A, /C, and /E (SNAP-25 and syntaxin) revealed that the BoNT/E cleavage site has the highest sequence conservation, while the cleavage site of type C has the least conservation, inversely correlating with the phenetic tree of BoNTs [125,133]. This observation suggests that there is a co-evolution between the neurotoxins and their substrates. 

The modular design of anthrax (PA, EF, and LF) and clostridial neurotoxins (heavy chain and light chain) suggested that these proteins have developed some remarkable machinery. There are still questions as to why they target different substrates, for example, clostridial neurotoxins only target the neuronal SNARE proteins whereas anthrax targets MAPKK as substrates. The conserved HEXXH motif, which is found in most metalloproteases, appeared as an ancient motif conserved from bacterial viral proteins (such as clostridial neurotoxin) to metalloproteases in human. Could the activation of neurotoxins through reduction of disulfide bond [95] between their heavy and light chains be an ancient form of activation of proenzyme for maturation of metalloproteases? By studying the bacterial toxins, such as clostridial neurotoxins, one can gain insight of their role in the evolutionary tree, the evolution of their counterparts in eukaryotes cells, and the fundamental basis of their functions.

Evolution is one of the greatest discoveries and widely debated topic in science. A few schools of thought hypothesized several possibilities for the origin of life [125], but the original primordial organism is missing in all current theories, it is “unknowable” and scientifically far from having definition. A root of any phenotypic tree is characterized as the most common ancestor, which is unknown. Even the RNA world hypothesis was not able to solve the “chicken – and - egg” problem. While defining these processes, one important fact is forgotten that all the organisms are made of molecule and there are several common molecules. These molecules take part in all reactions inside or outside of the cells. However, many of these reactions are same, such as those that release or store energy. Additionally, the processes are similar and rely on the exact same molecules. These molecules are widespread and termed as “molecular fossils”, such as ATP (adenosine tri phosphate). So, a more inclusive definition of evolution would include diversity in molecules. Diversity in species, organisms and biology comes later and can be defined as a subsequent state of molecular evolution. 

Internal motion and dynamics are directly associated with the function of any molecule and should be considered as an important parameter to drive evolution. These motions offer some extraordinary advantages to the molecule, such as multi-functionality (by providing large surface area for interaction), modification in structure and function, and in turn adaptability. 

Considering the above approach, bacteria and viruses are the oldest organisms, (*Clostridium* family is about 2.5 billion years old) and molecules carried by them are some of the oldest molecules. Even if they are highly potent (toxic) molecules, they are some of most remarkable systems produced by the nature. Therefore, they can be utilized as model molecules to redefine evolution. Definition of evolution should consider origin of molecular properties, such as atomic organization, molecular flexibility, folding/shape of different atoms/molecules, and more importantly, molecular motion (dynamics). As mentioned above, molecular flexibility and motion are very important characteristics which provide necessary energy to introduce any changes or modifications. Molecular motions could be the fundamental differences between different systems, although they have similar molecular shape/organization. 

Bacterial toxins are either multi-domain and/or complex proteins. Are they flexible? The answer to this question is as follows: The presence of multiple domains and complexing with other proteins require larger surface area for interactions, which in turn forces even less surface area available for intra-molecular interactions and makes them more likely to be flexible in isolation. Additionally, there is a correlation between flexibility and secondary structure of BoNTs. However, correlating flexibility with secondary structure could be a double-edged sword. For example, BoNT/A toxin consists of a binding domain (β-sheets), a translocation domain (α-helix), and a catalytic domain (α/β protein). This structural organization is a unique and self-explanatory; for binding molecules need rigidity, for translocation molecule needs fluidity (α-helix), and for catalysis molecule needs binding and dynamism (α-proteins tend to be more flexible than β-proteins [134]). Alternatives to the above view, cry proteins [98], three-finger proteins and spider toxins are having structural and functional divergence (β-proteins), resulting from the differential secondary structural organization of β-sheets and loops. So, fitting between flexibility and secondary structure is non-linear. It would be appropriate to conclude that the flexibility of any molecule is a result of fine play between different elements of structural organization and dynamics of the molecule certainly plays an important role in that. 

In general, bacterial evolution refers to the heritable genetic changes, which are accumulated from adaptation, and are associated with environmental changes or immune responses of the host. Because of short generation time and large population, they are the best candidates to study evolution. Additionally, protein toxins provide a very good tool to study this phenomenon. Is the creation of these nanomachines (widely referred as virulence factors) for defense or offense or kill? Apparently not so. The reasons for the above statement are: a) bacterial toxins add an extra energy burden for the bacterial cells (mostly because of their size and function), and b) they seem to divide labor between cells, mostly with only a fraction of cells specializing in toxin production (e.g., *C. botulinum* [135]). *C. botulinum* spores are ubiquitous, but the occurrence of botulism is meager. Thus, it is possible that *C. botulinum* grow and produce spores without necessarily producing toxins needed to kill, indicating the production of toxin may have function other than defense or to kill. Consequently, neurotoxins may have other roles in the ecological environment of *C. botulinum.*

A 2010 study found that a gene is present in the gut microbes of Japanese individuals but absent in the people from North America. Seaweeds are part of dietary staple of Japanese, and this gene is helpful in breaking down the carbohydrates from the seaweed, indicating some other forces are driving diversities among the same species and question central role of genes. Therefore, it is possible that some additional mechanisms are operational in the development of evolutionary traits. 

**Figure 4:**
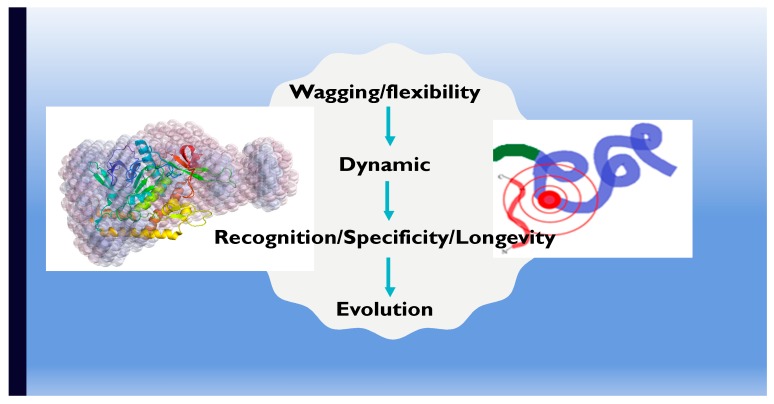
A schematic of relationship between structure-function of a bacterial protein and evolution. The differences between crystal and solution structure of a protein (on the left side), and role of flexibility in substrate recognition (on the right side) suggest us to hypothesize that the flexibility could be a parameter to define and direct evolution. The preliminary model, as suggested in this figure, involves unconventional steps to define evolutionary practices. This model brings shuttle molecular properties in the front, to define the fundamental steps of evolution rather than the gross structural and functional features.

Some may argue that these phenomena may be due to transmission of epigenetic markers that influence fertility, longevity, disease resistance, and other genetic inheritance. Who are the first responders to these epigenetic markers? Chemicals or molecules, in this case biological molecules. However, genes are not the first responders; it is the proteins and other biomolecules on the surface or inside the cells. These molecules are influenced by signals transmitted in the environment. These signals are created by dynamics of the ecosystem. The force or energy created by these ecological dynamics influences the dynamics of the biomolecules facilitating evolutionary processes. One of the ways to create an evolutionary correlation based on dynamics is to take advantage of the entropy concept. This will allow us to investigate the correlation between the target molecule and its possible interactions. Chang, Kumar, and Singh [125] employed a similar strategy to establish the correlation between botulinum toxin and its substrate. Entropy is a fundamental concept, however, and using this concept with biological molecules is a double-edged sword. Protein folding, function, organization of cells are some of the examples where the concept of entropy fails. Therefore, a more fundamental term, for creating diversity and allowing evolution, should be a primordial force which is driving evolution and entropy is just to introduce diversity in the evolutionary force. Figure 4 is a schematic of this explanation, which explains the role of dynamics in evolution of molecular structure and functions. This explanation is counterintuitive because this challenges the notion of folding as a primary goal. However, according to this explanation or hypothesis, function is the only goal and folding is not that important (IDPs are the best examples for this hypothesis).

In summary, bacterial toxins are one of the oldest molecules, which evolved as a virulence factor for the bacteria. Their structural and functional diversity makes it difficult to find the evolutionary lineage of these proteins. Although we have some neighboring relationship now to some of these toxins, research on finding the evolutionary lineage has several benefits. For finding the evolutionary lineage, we need to think out of the box, which allows us to rethink the current paradigm of evolutionary traits. Additionally, bacterial toxins, such as botulinum toxins, are the best model for examining this concept. They can provide a better understanding of predator-prey relationship, host pathogenesis, mimicry, transfer of molecular properties, and the fundamental biochemical understanding.

## Figures and Tables

**Table 1:** Biological activities of bacterial toxins.

Toxin	Biological Activity	Ref.
Cholera Toxin	Activation of adenylate cyclase; increasing intracellular cAMP, fluid and electrolytes secretion in intestinal epithelium leading to diarrhea	[5]
Shiga Toxin	Inactivates 60S ribosomal subunit, inhibition of protein synthesis	[6]
*E. coli* heat-labile toxin LT	Similar to cholera toxin	[7]
*E. coli* ST toxin	Binding to heat-stable enterotoxins (ST) to a guanylate cyclase receptor leading to an increase in cyclic GMP (cGMP), affect electrolyte reflux.	[8]
Diphtheria toxin	Inhibition of protein synthesis	[9]
Pseudomonas Exotoxin A	Inhibition of protein synthesis	[10]
Pertussis toxin	Adenylate cyclase inhibition, increase in the level of cAMP in phagocytes, affect on hormonal activity and reduction of phagocytic activity	[11]
Anthrax toxin	Induction of cytokine release and death of target cells	[12]
*Staphylococcus aureus* Exfoliating B	Separation of stratum granulosum of the epidermis	[13]
*Bordetella pertussis* AC toxin	Increase in cAMP in phagocytosis resulting in the inhibition of phagocytosis by neutrophilis and macrophages, also cause hemolysis and leukolysis	[14]
Perfringens enterotoxin	Stimulation of adenylate cyclase activity resulting in increase of cAMP in epithelial cells.	[15]
Staphylococcus enterotoxins	Immune system activation, including lymphocytes and macrophages	[16]
*Staphylococcus aureus alpha toxin*	Cell membrane pore formation	[17]
*Staphylococcus aureus* toxic shock syndrome toxin (TSST)	Action on the vascular system causing inflammation, fever and shock.	[18]
*Staphylococcus aureus* Erythrogenic toxin (SPE)	Similar to TSST	[19]
Botulinum Toxin	Inhibition of presynaptic acetylcholine release in PNS	[20]
Tetanus Toxin	Inhibition of neurotransmitter release in CNS	[21]

**Table 2:** Half-life t_1/2_ of antibodies, endogenous and exogenous proteins (including toxins). Antibodies are expected to be stable. For endogenous proteins, t_1/2_ depends on the cellular growth phase and types. For e.g., in human cancer cells t_1/2_ of endogenous proteins range from ~45 min. to ~22 h [96]. Toxins being an exogenous molecule live inside the target cells for longer than most of the proteins produced inside the cells.

Protein	t_1/2_ (Half-Life)
**Antibodies**	
Murine IgG2a	~8.4 days
Human IgG1 Fab fragment	~9 days
Human IgG1	~14 days
**Endogenous Proteins**	
Ornithine Decarboxylase	~11 min
Occludin	~2 h
Tyrosine Amino transferase	~3–4 h
Endogenous DISC1 protein	~6 h
Anti-apoptotic protein Bcl-2	~20 h
Na, K-ATPase 1	~40 h.
Arginase	~4 days
Albumin	~19 days
Nicotinamide adenine dinucleotide glycohydrolase	~20 days
**Bacterial Toxins**	
Staphylococcus enterotoxins	~2 h.
Cholera Toxin	~5 h
Shiga Toxin	~4 days
Diphtheria Toxin	>2 days
Tetanus Toxin	~5–6 days
Botulinum Toxin	~30–180 days
